# Spurious T3 Thyrotoxicosis Unmasking Multiple Myeloma

**DOI:** 10.1155/2013/739302

**Published:** 2013-07-29

**Authors:** Marianna Antonopoulou, Arnold Silverberg

**Affiliations:** ^1^SUNY Downstate Medical Center, Department of Endocrinology, 450 Clarkson Avenue, Box 1205, Brooklyn, NY 11203, USA; ^2^Maimonides Medical Center, Department of Endocrinology, 4802 Tenth Avenue Brooklyn, NY 11219, USA

## Abstract

*Objective*. To document a case of spurious T3 thyrotoxicosis in a 54-year-old woman. *Methods*. We present the diagnostic approach of a patient with euthyroid hypertri-iodothyronemia. *Results*. A 54-year-old, clinically euthyroid woman without personal or family history of thyroid disease referred to endocrinology for possible T3 thyrotoxicosis, after thyroid function tests revealed total T3 > 800 ng/dL (reference range 60–181), normal TSH, and T4. The laboratory data were not compatible with the clinical picture, so thyroid binding globulin abnormalities were suspected. Additional laboratory studies confirmed the diagnosis of multiple myeloma. *Conclusion*. Monoclonal gammopathy is characterized by the presence of a monoclonal immunoglobulin in the serum or urine, occurring in multiple myeloma, and can cause assay interference and spurious results. We identify a newly recognized cause of euthyroid hypertri-iodothyronemia, due to binding of T3 to monoclonal immunoglobulins in the setting of multiple myeloma. Our case is the only one to date suggesting that monoclonal immunoglobulins from multiple myeloma may exhibit binding to T3 only.

## 1. Introduction

Many conditions may interfere with the measurement of total T4 and T3, and may also cause small changes in free T3, and T4 levels. These conditions in the past were a diagnostic challenge, and patients may have been falsely treated for thyroid disease. Nowadays, TSH alone is considered a sufficient screening tool to rule out thyroid dysfunction, without being followed with a T3, T4 measurement [1]. But if T3 and T4 are measured and the patient is found to have abnormal levels of thyroid hormones, closer evaluation is needed.

## 2. Case Report

Our patient is a 54-year-old woman, who was referred from primary care physician for elevated T3 and possible T3 thyrotoxicosis. For the past few months, she had been complaining from fatigue and insomnia. She denied history of weight loss, hyperdefecation, heat intolerance, skin or hair changes, tremors, visual changes, and palpitations. She has no known personal or family history of thyroid disease. Physical exam revealed no palpable goiter, and the patient was clinically euthyroid. Previous thyroid function tests were all within normal limits. Patient's past medical history includes hypertension and total abdominal hysterectomy with bilateral salpingoophorectomy in 2006 for benign disease. Home medications include amlodipine 5 mg, irbesartan 150 mg, and hydrochlorothiazide 12.5 mg. After complaining of fatigue, her primary care physician ordered thyroid function tests, which showed TSH 2.35 *μ*IU/mL (reference range 0.545–4.784), T3 > 800 ng/dL (60–181), T4 6.3 ug/dL (4.5–10.9), free T4 0.94 ng/dL (0.8–1.8), and T3 uptake 36.2% (22.5–37).

 The previous thyroid function tests, using single antibody radioimmunoassay (RIA), were repeated with the same findings. Since TSH was within normal limits and not suppressed, we suspected TBG abnormalities. Additional laboratory studies revealed FT3 of 2.9 pg/mL (2.3–4.2), negative hepatitis panel, total protein of 12.3 mg/dL (6.2–8.3), albumin of 2.9 g/dL (3.6–5.3), globulin 9.4 g/dL (2.1–3.7), normal creatinine, calcium, and normocytic anemia. The high level of globulin raised suspicion for multiple myeloma, and serum protein electrophoresis showed gamma globulin of 6.41 g/dL (0.60–1.6) with an M spike, consistent with diagnosis of multiple myeloma.

Patient was clinically and biochemically euthyroid since TSH and free T3, T4 levels were within normal limits, but total T3 was spuriously elevated due to excess gamma globulin. Patient was urgently referred for hematological evaluation.

## 3. Discussion

The major iodothyronines are poorly soluble in water and therefore bind reversibly to plasma proteins. Both T4 and T3 are bound to one of three binding proteins, the thyroxine-binding globulin (TBG), transthyretin (thyroxine-binding prealbumin or TTR), and albumin. Approximately 99.97% of T4 and 99.7% of T3 are bound to these proteins. T3 is 80% bound to TBG, 5% to TTR, and 15% to albumin and lipoproteins [2]. Between 3% and 6% of plasma, T4 and T3 are bound to lipoproteins. This binding is of uncertain physiologic significance but can play a role in targeting T4 delivery to specific tissues [3]. The remainder is the hormonally active free hormone. The assays for total T3, T4 measure both free and bound hormones and this is why any change in these binding proteins will result in change in the serum concentration of T4 and T3, even though T4 and T3 production is not changed.

Euthyroid hyperthyroxinemia signifies that TSH is within normal limits and patient is clinically euthyroid, but total T4 or T3 is high with or without normal free thyroid hormone levels, and TBG abnormalities are frequent causes. TBG excess production can be hereditary, which is X-linked dominant transmission, or it can be secondary to excess estrogens, as in pregnancy, use of oral contraceptives, hormone replacement, and medications like raloxifene or tamoxifen [4, 5]. The more highly sialylated TBG is cleared more slowly from plasma than the more positively charged TBG, because sialylation inhibits the hepatic uptake of glycoproteins. Increased estrogen levels result in an increase of acidic bands of TBG, and thus decreased clearance and TBG excess. The latter can also be a sequel of acute or mostly in chronic active hepatitis, especially hepatitis C [6]. Several medications have been known to cause excess TBG levels, like 5-fluorouracil, clofibrate, and opiates [7–9]. Also, rarely, it has been described that acute intermittent porphyria is associated with high TBG levels [10].

Autoantibodies to T3 or T4 have also been described as thyroid hormone-binding proteins, causing falsely high or low levels [11, 12]. The prevalence of anti-T3 or anti-T4 antibodies among healthy individuals was found to be 1.8% by Sakata et al., but interference of the latter in the radioimmunoassay of free thyroid hormones was exceptional [13]. Usually, these antibodies are of polyclonal origin, but monoclonal antibodies have also been described [14–17].

Our patient was not on hormone replacement therapy or on any other medication that could alter TBG levels, and the hepatitis panel was negative. The patient had no clinical signs and symptoms of acute intermittent porphyria but had a negative family history of thyroid hormone abnormalities and normal previous thyroid function tests, making hereditary excess TBG production less likely. To date, there has been one case report describing factitious hyperthyroxinemia, due to a monoclonal IgA, in a case of multiple myeloma by Cissewski et al. in 1993. In this case report of a clinically euthyroid patient with IgA k myeloma, TSH and free T4 were normal, with high T3, T4. Further investigation showed that the IgA was acting as a high-capacity, low-affinity binding protein for T3 and T4, raising the total thyroid hormone levels but not altering the free hormone levels [18]. Increased binding of thyroid hormones by IgM or IgG has been described in a case of hypothyroidism in a patient with Waldenstrom's macroglobulinemia. Hypothyroidism was attributed to the binding of the thyroid hormones by IgG and IgM, which reduced T3 and T4 availability for the metabolic action at a tissue level [15]. 

In our case, serum protein electrophoresis was ordered, which confirmed high gamma globulin with an M spike. Free thyroid hormone levels were within normal limits, and only total T3 was elevated, suggesting that the patient was euthyroid and high T3 level was spurious. Our patient did not have any clinical evidence suggesting abnormal TBG, so the high total T3 is most likely attributable to the high monoclonal immunoglobulin level, binding T3 with high affinity but not T4, as shown by normal T4 levels. 

## 4. Conclusion

It is important to recognize that multiple myeloma is one of the causes of falsely elevated thyroid hormone levels. The monoclonal immunoglobulin can bind with T3 as suggested in our case and also T4 as shown by Cissewski et al., causing elevated total but normal free thyroid hormone levels, with a normal TSH. This is another cause of euthyroid hyperthyroxinemia, involving a newly recognized thyroid-binding protein. To date, there has been only one case report documenting euthyroid hyperthyroxinemia due to a monoclonal IgA binding T3 and T4, in the setting of multiple myeloma. However, in that case, both total T4 and T3 were elevated, and the affinity for T4 was 10 times higher compared to T3. Our case report is the only one to date suggesting that monoclonal immunoglobulins from multiple myeloma may exhibit binding with higher affinity to T3 only. 

## Abbreviations

IgA: Immunoglobulin A
RIA: Antibody radioimmunoassay
T3: Triiodothyronine
T4: Thyroxine
TBG: Thyroxine-binding globulin
TSH: Thyroide-stimulating hormone
TTR: Transthyretin or thyroxine-binding prealbumin.
